# It’s *Not* Just Lunch: Extra-Pair Commensality Can Trigger Sexual Jealousy

**DOI:** 10.1371/journal.pone.0040445

**Published:** 2012-07-11

**Authors:** Kevin M. Kniffin, Brian Wansink

**Affiliations:** Dyson School of Applied Economics and Management, Cornell University, Ithaca, New York, United States of America; Umeå University, Sweden

## Abstract

Do people believe that sharing food might involve sharing more than just food? To investigate this, participants were asked to rate how jealous they (Study 1) – or their best friend (Study 2) – would be if their current romantic partner were contacted by an ex-romantic partner and subsequently engaged in an array of food- and drink-based activities. We consistently find – across both men and women – that meals elicit more jealousy than face-to-face interactions that do not involve eating, such as having coffee. These findings suggest that people generally presume that sharing a meal enhances cooperation. In the context of romantic pairs, we find that participants are attuned to relationship risks that extra-pair commensality can present. For romantic partners left out of a meal, we find a common view that lunch, for example, is *not* “just lunch.”

## Introduction

“It’s Just Lunch” is the name of a matchmaking service that aims to attract potential subscribers with the idea that lunch provides a non-threatening environment to meet an unfamiliar person who shares interest to develop a romantic relationship. Of course, against the backdrop of studies that substantiate the importance of commensality – or eating together – within families [1], [2] and romantic pairs [3]–[5], it is reasonable to question whether a meal such as lunch is really just about lunch. In light of recognizing that commensality is part of the fabric of people’s most intimate relationships, it becomes clear that the practice of eating together might have functional significance beyond the concurrent consumption of calories.

Given that communal food procurement, preparation, and eating are considered quintessential human activities [6], it is interesting to recognize that modern technology – such as refrigerators and microwaves – and specialized businesses – such as restaurants and pizza delivery – have unbundled food procurement and preparation from consumption. Nevertheless, even though resources exist today to permit eating alone, it continues to be a normal practice for people to eat in groups [7], [8]. Focusing on romantic pairs, previous researchers have documented the importance of food for courtship and explored questions relating to specific preferences for type of cuisine, price, and home or restaurant locations [3]–[5].

In this paper, we explore the degree to which “extra-pair commensality” – eating without one’s current romantic partner with one or more other people – might elicit jealousy and whether it varies between men and women. While there are robust debates concerning the degree to which jealousy is an emotional adaptation that helps people guard against cheaters [9]–[12], the disagreements have focused on a general pattern whereby men appear to become more jealous about physical cheating and women tend to be more jealous about emotional cheating. Evolutionary psychologists contend that such a pattern makes sense since men – whose role in reproduction is less certain – would sensibly respond more to physical cheating to help ensure their paternity of any offspring whereas women tend to respond more to the diversion of attention or resources that might be entailed by emotional cheating [9].

Commensality is interesting to consider in this context since eating together involves physical and social components. Most generally, we apply a functional view of jealousy and hypothesize that if extra-pair commensality elicits relatively jealous reactions, then it suggests that people are evolved to recognize that eating together tends to involve, or perhaps lead to, something “more than food.” More specifically, our studies contribute new subtlety to debates concerning jealousy since our stimuli are not restricted to contrasts between physical and emotional affairs. For example, while evolutionary psychology predicts that men will tend to respond more strongly than women to their mates engaging in “extra-pair copulations” [13] – a term that is borrowed from biological field studies, our consideration of extra-pair commensality broadens the set of activities that might stimulate jealousy within romantic pair bonds. While we could have investigated the degree to which jealousy is elicited by other extra-pair activities such as night-club-dancing with someone other than one’s romantic partner, we focused on more mundane activities such as eating and drinking since people tend to eat and drink several times each day.

### Food Sharing and Social Behavior

While food consumption has been heavily studied in relation to physical outcomes such as weight gain [7], [8], it is relatively novel for close attention to be paid to the influence of food upon social behavior. Among the experiments that have explored this topic, Williams and Bargh [14] report that the receipt of warm beverages appears to elicit favorable perceptions. Less favorably, researchers have found that diet soda consumption appears to contribute to impulsiveness [15] and non-diet soda consumption appears – in a survey of urban high school students – to influence the rate of antisocial behaviors [16]. In a related observational study of judges, Danziger, Levav, and Avnaim-Pesso [17] found that judgments were significantly more lenient immediately following meal breaks in contrast with decisions that were issued immediately prior to meal breaks.

Our studies build upon previous research by extending the hypothesis that people regard the communal consumption of food to have functional significance for social relationships. More specifically, if commensality were regarded implicitly as a bonding mechanism, then we would expect that extra-pair commensality would trigger jealousy within romantic pairs. If commensality were regarded as nothing more than the concurrent, co-located consumption of food, then we would expect that extra-pair commensality would trigger as much jealousy as other forms of face-to-face interaction, such as meeting for coffee.

Moreover, if extra-pair commensality were regarded as something that is potentially threatening to one’s romantic relationship, then we can infer – if we accept a common model of sex-specific patterns of jealousy [9] – that men will react more strongly than women if eating together is viewed as physical and women will react more strongly if commensality is viewed as primarily emotional or social.

### Ethics Statement

For each of the studies that we conducted, participants provided informed consent orally since our commitment to conduct anonymous analyses did not require written consent. Participants acknowledged their understanding of the consent process through gestures and we reminded them that they could end their participation at any point during the study without penalty.

Our consent form and procedures were approved by the Cornell University Institutional Review Board (IRB). The use of verbal consent was approved by the Cornell University IRB and no written consent form was needed.

## Experiment 1

### Method

79 undergraduate students (52 males) at a private university in the Northeastern United States participated in this study in exchange for a nominal cash incentive. 96.2% of the respondents reported ages between 18 and 22 years old.

Participants were informed that “the next six questions ask you to imagine how you would react to a variety of hypothetical vignettes” and asked “Consequently, please use your imagination to respond as if the hypothetical event really happened.”

In randomized order, participants were presented with six vignettes that each started by noting that “Recently, your [romantic partner] was contacted by his/her ex-[romantic partner] and she/he spent approximately one hour” (1) corresponding via email, (2) talking on the phone, (3) meeting for late-morning coffee, (4) meeting for a late-morning meal (or Lunch), (5) meeting for late-afternoon coffee, and (6) meeting for a late-afternoon meal (or Dinner).

In order to personalize the vignettes, male participants were asked to rate how they would respond to hypothetical conditions involving their girlfriend engaging in communication with her ex-boyfriend. Likewise, female participants were asked to rate how they would respond to hypothetical conditions involving their boyfriend engaging in communication with his ex-girlfriend.

Participants were then asked for each question “On a scale of 1 to 5, please estimate how jealous you would be,” with 1 equal to “Not at all Jealous” and 5 equal to “Very Jealous.”

### Results

Remarkably, no significant sex differences existed for any of the conditions and we consequently report means in the upper row of Table 1 for the full sample. Unsurprisingly, participants estimated higher degrees of jealousy for direct communications. For example, Phone communications elicited significantly more jealousy than Email correspondence (*t* = −6.01, *p*<.001).

**Table 1:** Average Jealousy Ratings Estimated for Each Scenario (and Standard Deviations).

	Email Correspondence	Phone Conversation	Late Morning Coffee	Lunch	Late Afternoon Coffee	Dinner
**Self-Reported** **Jealousy [Study 1]**	2.92 (1.15)	3.37 (1.15)	3.33 (1.19)	3.49 (1.19)	3.51 (1.13)	3.57 (1.17)
**Best Friend’s** **Jealousy [Study 2]**	2.93 (1.15)	3.53 (1.13)	3.49 (1.19)	3.61 (1.15)	3.58 (1.24)	3.86 (1.16)

1 =  Not at all Jealous and 5 =  Very Jealous.

With respect to the four eating and drinking vignettes, Lunch elicited significantly more jealousy than Late Morning Coffee (*t * = −2.97, *p*<.01) and Late Afternoon Coffee sparked more jealousy than Late Morning Coffee (*t * = −3.49, *p*  = .001). When we collapsed the two coffee and meal variants, we find that Meals elicit significantly more jealousy than Coffees (*t*  = 2.16, *p*  = .034), Meals elicits more than Phone conversations (*t*  = 2.34, *p*  = .022), and Coffees do not elicit more jealousy than Phone conversations.

## Experiment 2

Independent from researchers arguing that jealousy might serve socially functional or adaptive purposes, common sentiments tend to regard jealousy as an undesirable trait [18], [19]. With this background, we conducted a second set of studies to address concerns about response bias by asking participants to estimate how their best friends would respond to the same set of conditions.

### Method

74 undergraduate students (51 females) at a private university in the Northeastern United States participated in this study in exchange for partial fulfillment of course credit and a nominal cash incentive. 59 participants were between 18 and 22 years old; 9 were 23 to 29 years old; 2 were 30 to 39; and, 4 were 40 or older.

In randomized order, participants were presented the same vignettes with the modification that people were asked to estimate how their “best same-sex friend” would respond if his or her romantic partner engaged in the six activities. As with Study 1, male and female participants each received sex-specific questions in which males were asked to estimate how their best male friend would respond if his girlfriend engaged in communications with her ex-boyfriend and female participants in the study were asked to estimate how their best female friend would respond if her boyfriend engaged in communications with his ex-girlfriend.

### Results

As with Study 1, we did not find sex differences for any condition and consequently we report the averages for our full sample in the lower row of Table 1. Consistent with response bias concerns that motivated us to conduct both studies, the average self-reported ratings are nominally higher for all six conditions. Likewise, we replicate the finding that Phone conversations elicits more jealousy than Email correspondence (*t*  = −4.90, *p*<.0001).

More specifically, Dinner draws significantly more jealousy than Late Afternoon Coffee (*t*  = 3.94, *p*<.0001) just as Dinner also elicits significantly more jealousy than Lunch (*t*  = .272, *p*<.01). Additionally, we find that Meals elicit more jealousy than Coffees (t  = 2.72, p<.01) and no significant differences exist between Meals and Phone or Coffees and Phone conversations.

## Discussion

**Figure 1:**
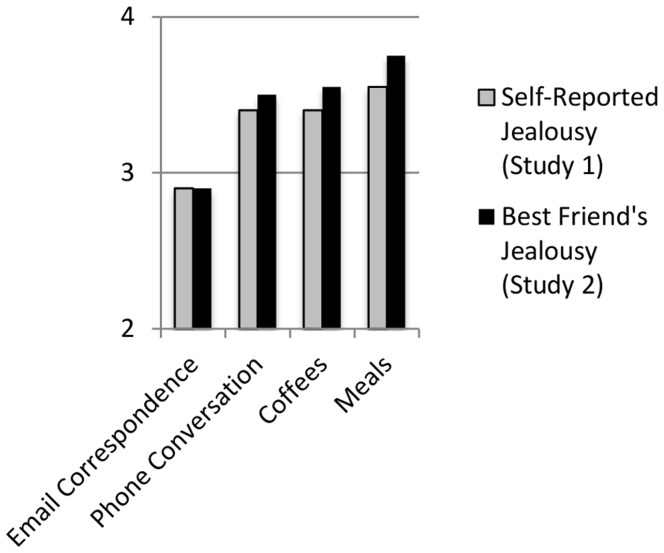
Average Jealousy Ratings Vary With the Social Context. When participants were asked to rate how jealous they (Study 1) or their best friend (Study 2) would be if their current romantic partner engaged in an array of activities with a former romantic partner, meals elicited significantly more jealousy than comparably long interactions involving coffee. Using a scale of 1 (Not At All Jealous) to 5 (Very Jealous), participants in both studies also reacted more strongly to direct communications when compared with email.

While our findings concerning Phone and Email communications are unsurprising, Figure 1 illustrates the interesting pattern whereby Meals consistently elicit more jealousy than face-to-face interactions (i.e., Coffees) that do not involve food. These findings suggest that people believe that commensality involves more than the physical consumption of calories. More specifically, the pattern across both studies suggests that people are attuned to the potential relationship threat that they implicitly expect can be posed by extra-pair commensality.

Against the backdrop of previous studies concerning jealousy, the absence of sex differences is notable. Given the importance of understanding jealousy in relation to aggression [11] and given previous studies that have highlighted sex differences, our findings of common attitudes about commensality are helpful. In particular, we can provisionally infer from our studies that people view commensality as an interaction that involves a mix of physical and emotional exchanges.

Against the backdrop of studies that treat cooperation as a puzzle that requires explanation [20]–[23], our findings highlight a mechanism – commensality – that has been relatively understudied as a tool for developing and strengthening social relationships. For example, while the existence of heterosexual romantic relationships poses no puzzle to evolutionary psychologists, our studies highlight a candidate mechanism for researchers seeking to understand why genetically unrelated non-kin competitors often opt to cooperate with each other [20]–[23]. Among other potential domains where commensality might be closely studied as a mechanism for community building, Wilson, Kauffman, and Purdy [24] identify communal eating of meals in a special high school for at-risk students as part of a success-generating cultural environment.

Of two primary limitations with our studies, the first involves our reliance on ratings from relatively homogenous samples of participants – undergraduate students enrolled at a private university in the Northeastern United States. As a consequence, future studies conducted with more heterogeneous groups of people will be necessary to test the generalizability of our findings. For example, it is plausible that different patterns would emerge if these questions were presented to different subgroups within the United States and, more broadly, different cultural groups across the globe where values related to meals and jealousy are certain to vary [25].

A second limitation of our studies involves the fact that our stimuli consistently presented the prospect of one’s current romantic partner eating, drinking, or communicating with a former romantic partner. Future studies will need to test the extent to which comparable patterns might exist when one’s current romantic partner eats, drinks, or communicates with a potential romantic partner with whom there is no history of romance. For example, it should not be assumed that jealousy would be elicited if a person learned that their romantic partner ate lunch with a newly hired co-worker and it is unlikely that jealousy would be elicited if their romantic partner ate with someone such as a significantly older widow or widower who lives next door and does not fit the profile of a potential romantic rival.

Beyond recommending research that addresses limitations of this paper, our focus on the influence of eating on the nature of social relationships opens several new lines of study. For example, given previous research that shows the importance of non-physical traits upon perceptions of physical attractiveness [26], it seems plausible that strangers who eat with each other might develop enhanced perceptions of each other’s physical attractiveness after sharing a meal. In fact, such a pattern is the kind of evidence that would validate the jealousy elicited in our studies as functional or adaptive responses to a relationship threat. More basically, while our studies did not find a difference between men and women with respect to jealousy, it seems plausible that pregnant women might demonstrate more jealousy of their partner’s extra-pair commensality if they are especially sensitive to the potential diversion of attention and resources to another person. Similarly, it might be the case that partners who are married tend to be more jealous – perhaps especially when the couple has children – when meals are shared outside of the pair bond just as it might be true that less jealousy is elicited when both partners are employed outside the home and, consequently, less dependent on each other’s income.

Most generally, our current findings contribute to growing interest concerning the influence of food upon individual and social behavior. While the relative homogeneity of our samples limits the degree to which we can draw broad generalizations, our studies suggest that the professional match-making company “It’s Just Lunch” perhaps unknowingly benefits from implicit beliefs about eating together that helps them to connect people. But moreover, our findings also suggest that a more accurate and innocuous saying might be “It’s Just Coffee” since people seem to view drinking coffee during the day as relatively more platonic.
